# Identifying Translational Longevity Targets With Genetically Mediated Transcriptome-Wide Association Studies

**DOI:** 10.1093/geroni/igaa057.3129

**Published:** 2020-12-16

**Authors:** Daniel Evans, Steven Cummings, Nicholas Schork

**Affiliations:** 1 California Pacific Medical Center, San Francisco, California, United States; 2 Translational Genomics Research Institute, Phoenix, Arizona, United States

## Abstract

We hypothesized that trait associations with genetically mediated gene expression could be used to screen for genes that are good candidates for translational studies of longevity. We compiled a collection of genetically-mediated transcriptome-wide association studies using 33 traits and outcomes from large-scale, publicly-available GWAS meta-analysis results. The traits/outcomes were grouped within eight categories (aging, anthropometric, cardiovascular, inflammation, lung function, metabolic, musculoskeletal, and neurological). To test the utility of this approach, we examined trait associations with the drug target of statins, and we correctly identified known therapeutic effects and adverse events of statins. Specifically addressing the hypothesis, we examined a collection of candidate longevity-associated genes and identified one gene associated with lifespan that appears to also be associated with protection from atrial fibrillation and hearing impairment without being associated with adverse events. This screening approach can be used to prioritize gene targets for longevity translational efforts.

